# The art of seeing the elephant in the room: 2D embeddings of single-cell data do make sense

**DOI:** 10.1371/journal.pcbi.1012403

**Published:** 2024-10-02

**Authors:** Jan Lause, Philipp Berens, Dmitry Kobak

**Affiliations:** 1 Hertie Institute for AI in Brain Health, University of Tübingen, Tübingen, Germany; 2 Tübingen AI Center, University of Tübingen, Tübingen, Germany; 3 IWR, Heidelberg University, Heidelberg, Germany; University of Virginia, UNITED STATES OF AMERICA

## Abstract

A recent paper claimed that *t*-SNE and UMAP embeddings of single-cell datasets are “specious” and fail to capture true biological structure. The authors argued that such embeddings are as arbitrary and as misleading as forcing the data into an elephant shape. Here we show that this conclusion was based on inadequate and limited metrics of embedding quality. More appropriate metrics quantifying neighborhood and class preservation reveal the elephant in the room: while *t*-SNE and UMAP embeddings of single-cell data do not preserve high-dimensional distances, they can nevertheless provide biologically relevant information.

In single-cell genomics, researchers often visualize data with 2D embedding methods such as *t*-SNE [1,2] and UMAP [3,4]. Chari and Pachter [5] criticize this practice: They claim that the resulting 2D embeddings fail to faithfully represent the original high-dimensional space, and that instead of meaningful structure these embeddings exhibit “arbitrary” and “specious” shapes. While we agree that 2D embeddings necessarily distort high-dimensional distances between data points [6,7], we believe that UMAP and *t*-SNE embeddings can nevertheless provide useful information. Here, we demonstrate that UMAP and *t*-SNE preserve cell neighborhoods and cell types, and that the conclusions of Chari and Pachter [5] are based on inadequate metrics of embedding quality.

To illustrate their point that *t*-SNE and UMAP embeddings are arbitrary, Chari and Pachter [5] designed Picasso, an autoencoder method that transforms data into an arbitrary predefined 2D shape, e.g., that of an elephant. The authors then compared four kinds of embeddings: the purposefully arbitrary elephant embedding, 2D PCA, *t*-SNE, and UMAP (Fig 1). For this, they used two metrics of embedding quality, both requiring class annotations: *inter-class correlation* measuring how well high-dimensional distances between class centroids are preserved in the 2D embedding and *intra-class correlation* measuring how well class variances are preserved. They found that across three scRNA-seq datasets, 2D PCA performed the best on those metrics, while the elephant embedding scored similar to or better than UMAP and *t*-SNE. We reproduced and confirmed these results (Fig 2A–2B).

**Fig 1:**
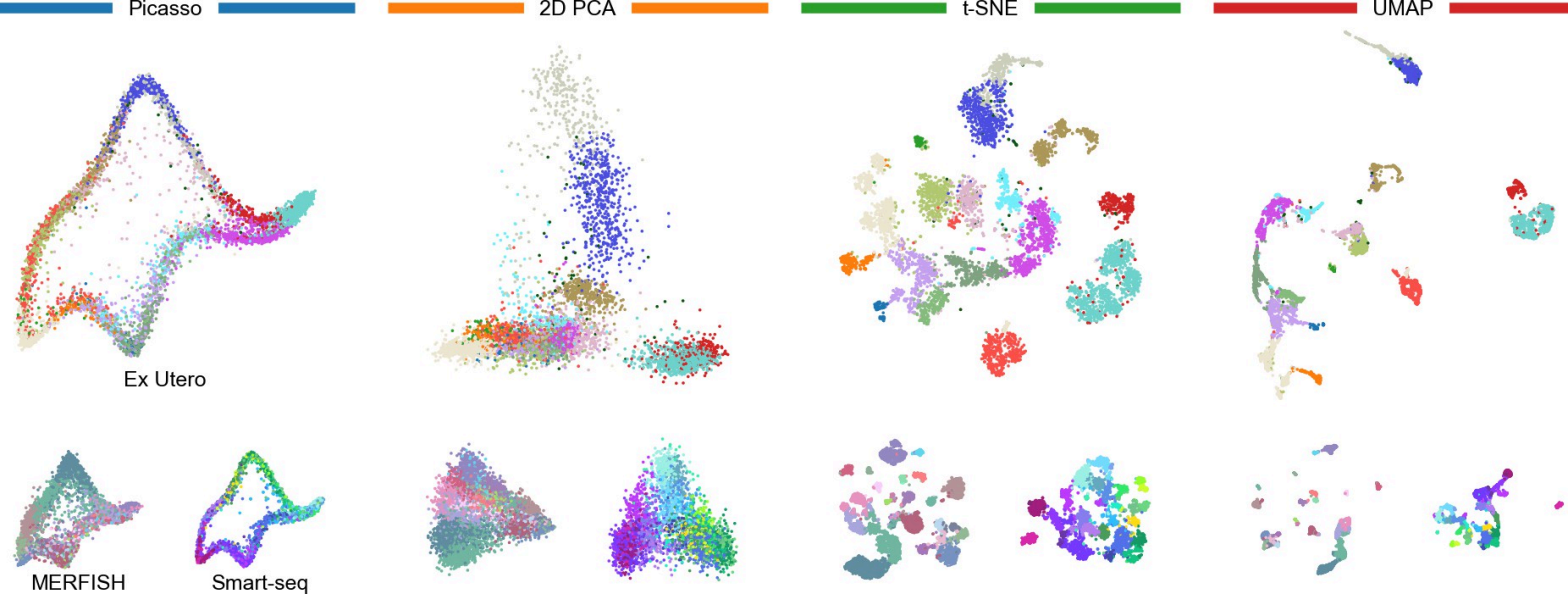
Evaluated embeddings. Large panels: Ex Utero dataset. Small panels: MERFISH and Smart-seq datasets. Colors correspond to cell types and are taken from Chari and Pachter [5]. See S1 Text for details.

**Fig 2:**
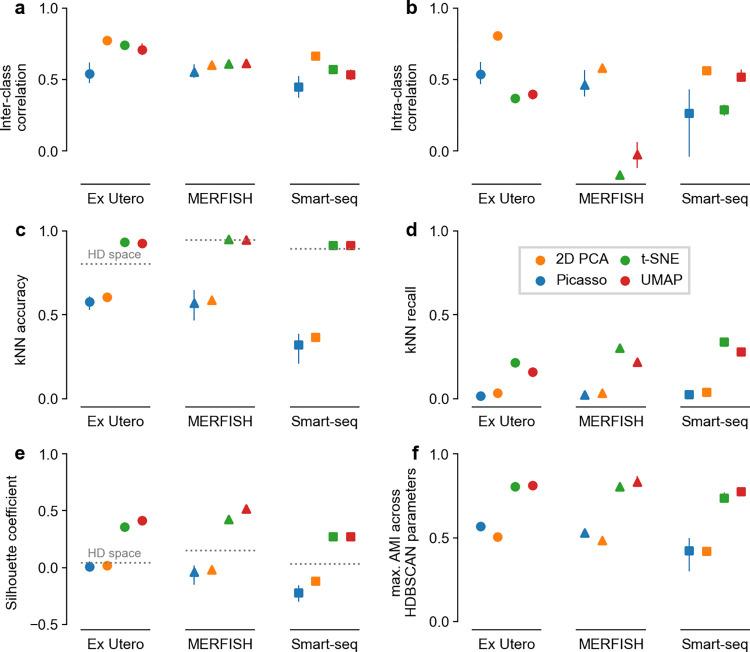
Embedding quality metrics. Panels correspond to metrics, colors correspond to embedding methods, marker shapes correspond to datasets. Averages over five runs, error bars go from the minimum to the maximum across runs. Dotted horizontal lines show the values of the metrics in the high-dimensional gene space. **a–b:** The two metrics from Chari and Pachter [5], reproducing the results from their Fig 7**C–**7**D:** *k*NN accuracy and *k*NN recall (*k* = 10). **e:** Silhouette coefficient. **f:** Maximum adjusted mutual information between classes and 2D clusters obtained with HDBSCAN using a range of hyperparameter values.

According to the authors, this means that *t*-SNE and UMAP are as arbitrary and as misleading as the Picasso elephant. Most online discussions and debates about their paper, including posts by the authors themselves, have prominently featured this argument and the powerful elephant metaphor to argue that “it’s time to stop making *t*-SNE & UMAP plots” [8]. In this Comment, we focus exclusively on this argument and do not discuss the rest of the Chari and Pachter [5] paper.

We believe that this argument is faulty because the metrics used by Chari and Pachter [5] are insufficient and only quantify a single aspect: both metrics focus on preservation of *distances*, where 2D PCA was unsurprisingly the best. But there is more to embeddings than distance preservation. It is visually apparent in the resulting embeddings that *t*-SNE and UMAP separate cell types, while 2D PCA and Picasso elephant lead to strongly overlapping types (Fig 1), but neither of the two metrics quantified that. Biologists are often interested in cell clusters, and so preservation of cell neighborhoods and visual separation of meaningful cell groups are important properties of 2D embeddings.

To quantify these aspects neglected by Chari and Pachter [5], we used four additional metrics, commonly employed in benchmark studies [9–11]: *k*-nearest-neighbor (*k*NN) accuracy, *k*NN recall [12], the silhouette coefficient [13], and the adjusted mutual information (AMI) between clusters and class labels [14].

The *k*NN accuracy quantifies how often the 2D neighbors are from the same class, while the *k*NN recall quantifies how often the 2D neighbors are the same as the high-dimensional neighbors. In both metrics, UMAP and *t*-SNE consistently and strongly outperformed PCA and Picasso elephant embeddings (Fig 2C–2D, >90% vs. <62% accuracy, >15% vs. <5% recall for all datasets). Even though the *k*NN recall was below 40% for all methods (Fig 2D), *k*NN accuracy was always above 90% for both UMAP and *t*-SNE (Fig 2C). This means that even though UMAP and *t*-SNE are not able to preserve high-dimensional nearest neighbors exactly, the low-dimensional neighbors tend to be from a close vicinity in the high-dimensional space, have the same cell type, and hence allow reliable *k*NN classification. In contrast, 2D PCA and the Picasso elephant fail at that.

The silhouette coefficient and the AMI both evaluate to what extent cell types appear as isolated islands in 2D. Specifically, the silhouette coefficient measures how compact and separated the given classes are in 2D, while the AMI evaluates how well clustering in 2D recovers the classes. In both metrics, *t*-SNE and UMAP strongly outperformed 2D PCA and Picasso elephant embeddings (Fig 2E–2F, >0.3 difference in silhouette score, >0.25 difference in AMI), in agreement with the visual impression (Fig 1).

The *k*NN accuracy and the silhouette coefficient can also be computed directly in the high-dimensional gene space. We found that *t*-SNE and UMAP showed similar or higher *k*NN accuracy and much higher silhouette coefficient than the original high-dimensional space (Fig 2C and 2E). This suggests that high-dimensional distances suffer from the curse of dimensionality, and that it may in fact be undesirable to preserve them in 2D visualisations. Indeed, single-cell biologists rarely use multidimensional scaling (MDS), an embedding method explicitly designed to preserve distances, because MDS often fails to represent the cluster structure in the data. This further underscores why using only distance-preservation metrics, as Chari and Pachter [5] did, is misguided.

All presented metrics except *k*NN recall rely on class labels, and our analysis, following Chari and Pachter [5], used labels derived in original publications via clustering. Therefore, these labels do not necessarily correspond to biological ground truth, and could potentially lead to biased comparisons. To address this concern, we used negative binomial sampling based on the Ex Utero dataset to simulate a dataset with known ground truth classes. Analyzing this simulated dataset gave the same conclusions: 2D PCA scored the best in the distance-based correlation metrics of Chari and Pachter [5], but only *t*-SNE and UMAP could separate the true classes, while Picasso and 2D PCA failed at that (Fig A in S1 Text).

Taken together, our results point to the elephant in the room: Even though they are not designed to preserve pairwise distances, *t*-SNE and UMAP embeddings are not arbitrary and do preserve meaningful structure of single-cell data, especially local neighborhoods and cluster structure. Claiming that Picasso and *t*-SNE/UMAP are “quantitatively similar in terms of fidelity to the data in ambient dimension” [5] is wrong. They are not.

That said, we do agree with Chari and Pachter [5] that 2D visualisations distort distances and should not be blindly trusted. Moreover, as Chari and Pachter [5], we do not recommend to use 2D embeddings for quantitative downstream analysis. However, paraphrasing George Box [15], we can say that *all 2D embeddings of high-dimensional data are wrong*, *but some are useful*. Indeed, one can use 2D embeddings to form hypotheses about the data structure, ranging from data quality control and sanity-checking of any algorithmic output, to more general hypotheses about cluster separability, relationships between adjacent clusters, or presence of outlying clusters. Of course, any generated insight should then be validated in the high-dimensional data by other means. Here, our conclusion differs strongly from that of Chari and Pachter [5]: while they claim that UMAP and *t*-SNE are “counter-productive for exploratory […] analyses”, we endorse them for that very purpose.

## Supporting information

S1 Text Supplementary Methods and Supplementary Figures. (PDF)
