# Supplementary material for: The art of seeing the elephant in the room: 2D embeddings of single-cell data do make sense
Source: PLoS Comput Biol. 2024 Oct 2;20(10):e1012403. doi: 10.1371/journal.pcbi.1012403 (PMC11446450; doi:10.1371/journal.pcbi.1012403)
Supplement: S1 Text — (PDF) [file pcbi.1012403.s001.pdf]

# Supplementary Methods

**Datasets and preprocessing** We used the same datasets as Chari and Pachter [1] (Table A) and followed the same pre-processing steps. The `Ex Utero` data was already log-normalized. We filtered empty genes and cells, and selected the 2 000 most highly variable genes (HVGs) with `scanpy.pp.highly_variable_genes()` with default settings [2]. The `MERFISH` data was already normalized, and we only performed the `log1p()` transform. The `Smart-seq` data was already log-normalized and had HVGs selected, so we used it as is. Chari and Pachter [1] additionally performed a standardization step on all datasets, which we omitted for simplicity, as it did not change the result qualitatively (see our Github repository for a direct comparison). Despite small differences in pre-processing choices, we obtained qualitatively very similar results in Fig 2a–b to what the original authors reported in their Fig 7.

| Name | Cells | Genes | Classes | Source | Count matrix | Metadata |
|---|---|---|---|---|---|---|
| `Ex Utero` | 6 205 | 19 588 | 19 | Aguilera-Castrejon et al. [3] | `normalized.assay85` | `MetaData.85` |
| `MERFISH` | 6 963 | 254 | 25 | Zhang et al. [4] | 10.22002/D1.2064 | 10.22002/D1.2063 |
| `Smart-seq` | 3 850 | 1 999 | 28 | Kim et al. [5] | 10.22002/D1.2071 | 10.22002/D1.2067 |

**Table A: Datasets.** `Ex Utero`: Files in GEO accession GSE149372. `MERFISH` and `Smart-seq`: DOIs.

**Simulation** We used negative binomial sampling to obtain a simulated version of the `Ex Utero` dataset with known ground-truth classes. For each cluster and each gene $g$ in the original dataset, we computed the proportion $p_g$ of UMI counts of this gene among all UMI counts in the cluster. For each cell $c$ belonging to this cluster in the original data, we then sampled new counts $X_{cg} \sim \mathrm{NB}(\mu = n_c p_g, \theta = 10)$, where $n_c$ is the cell's original total UMI count. Overdispersion parameter $\theta = 10$ leads to some additional variance compared to the Poisson distribution. This procedure preserved the number of genes, the number of cells, and all class abundances, and ensured realistic marginal distributions of simulated counts per cell and per gene. The counts of each simulated gene in each class followed an independent negative binomial distribution around the gene's mean expression in the original `Ex Utero` cluster. Finally, we performed the same pre-processing as above on the simulated counts (depth normalization, scaling normalized counts to 10 000 counts per cell, `log1p()` transform, `scanpy` default HVG selection).

**Embeddings** We used the high-dimensional gene space after pre-processing and gene selection as input to all embedding methods. For the elephant embeddings, we used the original Picasso code by Chari and Pachter [1] with minimal adjustments needed to provide the random seed for reproducibility (`https://github.com/berenslab/picasso`). We ran Picasso for 500 epochs with default settings. For PCA, we used `scikit-learn 1.3.0` [6] with default parameters. For $t$-SNE and UMAP, we followed Chari and Pachter [1] and first reduced the pre-processed count matrices to 50 dimensions with PCA and used that as input to `openTSNE 1.0.1` [7] and `umap-learn 0.5.5` with default parameters. The 50-dimensional PCA was used in no other part of the analysis. In all plots, we used the class labels and colors from Chari and Pachter [1], except for minor adjustments to the `Ex Utero` colors, where we introduced four additional colors to make all classes discernible.

**Embedding quality metrics** Following Chari and Pachter [1], we computed their intra- and inter-class correlation metrics using both $L^1$ and $L^2$ distances (see our Github repository for a direct comparison). As we did not observe qualitative differences between the two variants, we only showed $L^2$ results here, and also used $L^2$ distances for all other metrics.

For $k$NN accuracy, we used the $k$ nearest neighbors in the 2D embedding to predict the class of each cell with a majority vote (this is essentially a leave-one-out cross-validation procedure). We reported raw accuracy here, but class-balanced accuracy gave qualitatively the same results (see our Github repository). For $k$NN recall, we computed (for each cell) the fraction of the $k$ nearest neighbors in the 2D embedding that are also among the $k$ nearest neighbors in the high-dimensional space. For both $k$NN metrics, we used $k = 10$, and averaged over all cells.

For the maximum AMI metric, we ran HDBSCAN [8] from `scikit-learn` on each embedding for nine hyperparameter values $\texttt{min\_samples} = \texttt{min\_size\_clusters} \in \{5, 10, 15, 20, 30, 40, 50, 75, 100\}$. All points that HDBSCAN left unclustered (noise points) we assigned to their nearest clusters. We then computed the adjusted mutual information (AMI) between each HDBSCAN result and the given cell type class labels, and picked the largest AMI. This way, the best performing hyperparameter was chosen for each embedding and each dataset.

The silhouette coefficient of each cell is defined as $(b - w)/\max(b, w)$ where $w$ is the average distance to cells from the same class and $b$ is the average distance to cells in the nearest other class. The silhouette coefficient is then averaged across all cells. We used `scikit-learn` to find $k$NNs, and to compute AMI and silhouette coefficients.

For all metrics that required a high-dimensional reference space for comparison (inter-class and intra-class correlations, $k$NN recall), we used the same high-dimensional gene space that we used as input to the embedding methods.

**Code** Our code in Python is available at https://github.com/berenslab/elephant-in-the-room.

## Supplementary Figures

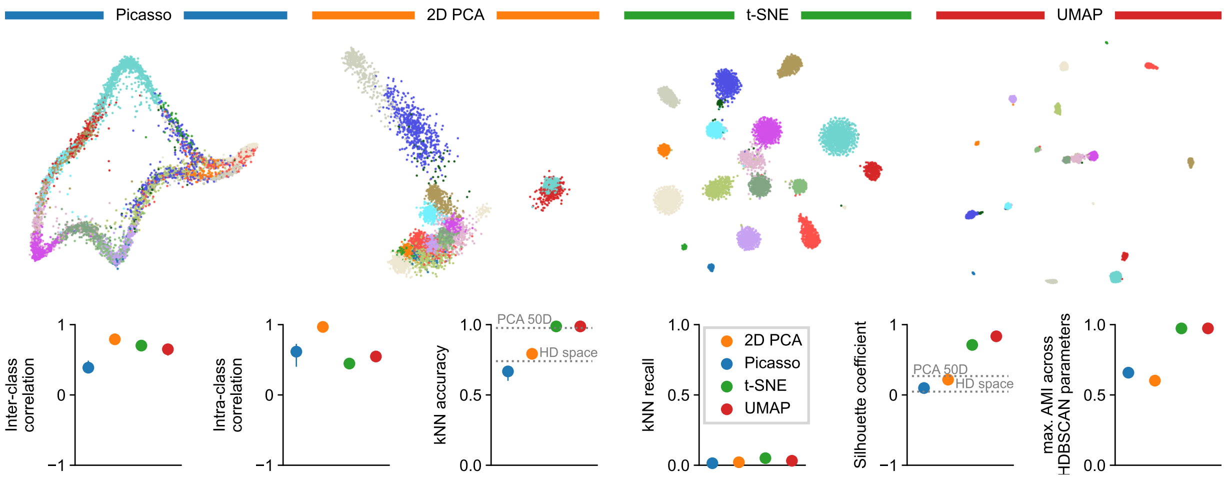


**Fig A: Simulated dataset with ground truth labels.** Simulation was based on the `Ex Utero` dataset and generated 19 classes using negative binomial sampling (see Supplementary Methods for details). Top row: Embeddings as in Fig 1. Bottom row: Embedding quality metrics as in Fig 2. The $k$NN recall values are very low because simulated classes do not have any internal structure. Dotted horizontal lines show the $k$NN accuracy and silhouette score in the high-dimensional gene space ("HD space") and the 50-dimensional PCA space ("PCA 50D").

## References


1. Tara Chari and Lior Pachter. The specious art of single-cell genomics. *PLOS Computational Biology*, 19(8):e1011288, 2023.

2. F Alexander Wolf, Philipp Angerer, and Fabian J Theis. SCANPY: large-scale single-cell gene expression data analysis. *Genome Biology*, 19:1–5, 2018.

3. Alejandro Aguilera-Castrejon, Bernardo Oldak, Tom Shani, Nadir Ghanem, Chen Itzkovich, Sharon Slomovich, Shadi Tarazi, Jonathan Bayerl, Valeriya Chugaeva, Muneef Ayyash, et al. Ex utero mouse embryogenesis from pre-gastrulation to late organogenesis. *Nature*, 593(7857):119–124, 2021.

4. Meng Zhang, Stephen W Eichhorn, Brian Zingg, Zizhen Yao, Kaelan Cotter, Hongkui Zeng, Hongwei Dong, and Xiaowei Zhuang. Spatially resolved cell atlas of the mouse primary motor cortex by merfish. *Nature*, 598(7879): 137–143, 2021.

5. Dong-Wook Kim, Zizhen Yao, Lucas T Graybuck, Tae Kyung Kim, Thuc Nghi Nguyen, Kimberly A Smith, Olivia Fong, Lynn Yi, Noushin Koulena, Nico Pierson, et al. Multimodal analysis of cell types in a hypothalamic node controlling social behavior. *Cell*, 179(3):713–728, 2019.

6. Fabian Pedregosa, Gaël Varoquaux, Alexandre Gramfort, Vincent Michel, Bertrand Thirion, Olivier Grisel, Mathieu Blondel, Peter Prettenhofer, Ron Weiss, Vincent Dubourg, et al. Scikit-learn: Machine learning in Python. *The Journal of Machine Learning Research*, 12:2825–2830, 2011.

7. Pavlin G Poličar, Martin Stražar, and Blaž Zupan. openTSNE: A modular python library for t-SNE dimensionality reduction and embedding. *Journal of Statistical Software*, 109:1–30, 2024.

8. Leland McInnes and John Healy. Accelerated hierarchical density based clustering. In *2017 IEEE International Conference on Data Mining Workshops (ICDMW)*, pages 33–42. IEEE, 2017.
